# Oxaliplatin resistance is enhanced by saracatinib via upregulation Wnt-ABCG1 signaling in hepatocellular carcinoma

**DOI:** 10.1186/s12885-019-6480-9

**Published:** 2020-01-13

**Authors:** Xia Liao, Ge Song, Zihan Xu, Yang Bu, Fan Chang, Fengan Jia, Xuelian Xiao, Xuejiao Ren, Mei Zhang, Qingan Jia

**Affiliations:** 1 Department of Nutrition, First Affiliated Hospital of Xi’an Jiaotong University, Xi’an, 710061 China; 2 Department of Burns and Plastic Surgery, Shaanxi Provincial People’s Hospital, Xi’an, 710068 China; 3 Department of Hepatobiliary Surgery, General Hospital, Ningxia Medical University, Yinchuan, 750001 China; 4 Metabolite Research Center, Shaanxi Institute of Microbiology, Xi’an, 710043 China; 5 Department of Hepatobiliary Surgery, First Affiliated Hospital of Xi’an Jiaotong University, 277 West Yanta Road, Xi’an, 710061 China; 6 Medical College of Yan’an University, Yan’an, 716000 China

**Keywords:** Hepatocellular carcinoma, Oxaliplatin resistance, Saracatinib, Wnt signaling, ABCG1

## Abstract

**Background:**

Chemo-resistance in hepatocellular carcinoma (HCC) is a major problem, and acquired drug resistance prevents cancer therapies from achieving complete responses. Molecular targeting therapy presents an opportunity to impede tumor through combination or sequential therapy, while the accurate effect is vague.

**Methods:**

The efficacy of combinations between oxaliplatin and anti-cancer molecular targeting drugs was screened. Strangely, the combined chemotherapy with oxaliplatin and saracatinib induced significantly antagonistic effects. Then the antitumor effects of combined treatment with saracatinib and oxaliplatin were confirmed in wide type HCC as well as in saracatinib- and oxaliplatin-resistant HCC. RNA sequencing was used to explore the resistance mechanism, and the roles of ATP-binding cassette transporter *G1* (*ABCG1)* and Wnt signaling in oxaliplatin resistance were confirmed.

**Results:**

Chemotherapy with oxaliplatin and saracatinib individually induced strong anti-HCC effects, while combined or sequential treatment of HCC cells with these two drugs exhibited reduced efficacy compared to treatment with the single drugs. And it was saracatinib treatment caused oxaliplatin resistance. RNA sequencing revealed 458 genes that were altered by treatment with saracatinib and oxaliplatin. Of these, the gene encoding *ABCG1* and Wnt-associated genes were significantly upregulated. Upregulation of *ABCG1* and oxaliplatin resistance were associated with activation of Wnt signaling. Interference with *ABCG1* expression or inhibition of Wnt signaling resulted in reversal of the saracatinib-induced oxaliplatin resistance in HCC.

**Conclusions:**

These studies demonstrated that combined or sequential chemotherapy with oxaliplatin and saracatinib reduced antitumor efficacy, and this antagonism was attributed to the activation of Wnt signaling and upregulation of *ABCG1* by saracatinib.

## Background

In clinical practice, more than 70% of patients with HCC are diagnosed at an advanced stage and are treated with a non-radical surgical regimen, including transcatheter arterial chemoembolization (TACE) and systemic chemotherapy [1]. Oxaliplatin has commonly been used, although the efficacy of oxaliplatin for HCC is poor, due to the presence of both intrinsic and acquired resistance. Oxaliplatin resistance in HCC is a major medical problem, and methods for improvement of the response to this chemotherapeutic are urgently needed [2]. Molecular targeting therapy presents a therapeutic opportunity to impede tumor relapse and reverse drug resistance, while the accurate combined effect is not yet clear in HCC.

In this study, the efficacy of combinations between oxaliplatin and anti-cancer molecular targeting drugs was screened, and saracatinib treatment actually induced resistance to oxaliplatin treatment was proved. Then we evaluated the response of HCC to oxaliplatin and saracatinib in vitro and in vivo, and RNA sequencing revealed that the antagonistic relationship between saracatinib and oxaliplatin stemmed from activation of the Wnt signaling pathway, resulting in increased expression of the ATP-binding cassette transporter *G1 ABCG1*. Finally, we proved interference with *ABCG1* expression or inhibition of Wnt signaling resulted in reversal of the saracatinib-induced oxaliplatin resistance in HCC. These findings indicate that combination or sequential therapy with oxaliplatin and saracatinib have negative effects on HCC via upregulation Wnt-ABCG1 signaling.

## Methods

### Cell lines and animals

Human HCC cell lines MHCC97L, which has high metastatic potential (established at Fudan University, Shanghai, China; RRID: CVCL_4973), and Hep3B, which has low metastatic potential (American Type Culture Collection, Rockville, MD, USA; RRID: CVCL_0326), were obtained from the Liver Cancer Institute of Fudan University (Shanghai, China). All cells were maintained in Dulbecco’s Modified Eagle’s Medium (DMEM; GIBCO, Grand Island, NY, USA) and supplemented with 10% fetal bovine serum (FBS; GIBCO) at 37 °C in a humidified incubator with 5% CO_2_. Cells were routinely screened for the presence of mycoplasma (Mycoplasma Detection Kit, Roche Diagnostics, Indianapolis, IN, USA).

Male BALB/c nu/nu mice (aged 4–6 weeks and weighing approximately 20 g) were obtained from the Chinese Academy of Science (SLRC, Shanghai, China) and raised in a controlled environment with 25 °C under standard pathogen-free conditions and a natural light/dark cycle (morning 8:00; afternoon 8:00), and were provided with water and standard diet. Animal protocols were approved by the ethics committee on Experimental Animals of Xi’an Jiaotong University.

### Reagents and antibodies

Oxaliplatin, and Src inhibitor saracatinib (AZD0530) were used for the construction of drug-resistant cell lines, and other anti-cancer molecular targeting drugs were purchased from ApexBio (Houston, TX, USA) and Selleck (Houston, TX, USA). Monoclonal antibodies to the following proteins were used in western blot: E-cadherin, vimentin, PCNA, FZD8, DKK1, AXIN2, WNT6, and β-catenin (purchased from Abcam, Cambridge, MA, USA) and p-LRP6, GSK-3β, AXIN2, cyclin D1, SRC, OCT4, ABCG1, and BCL-2 (purchased from Proteintech, Chicago, IL, USA).

### In vitro drug sensitivity assay

MHCC97L cells were seeded in 96-well plates at 2500 cells per well. Twelve hours after plating, cells were treated with anti-cancer molecular targeting drugs library (including 29 inhibitors in PI3K, MAPK signaling et al). After 72 h of incubation at 37 °C in a 5% CO2 humidified incubator, cell viability was analyzed using Cell Counting Kit 8 (CCK8; Dojindo, Gaithersburg, MD, USA). The drugs were stored and diluted according to the manufacturers’ instructions.

### Generation of oxaliplatin- and saracatinib-resistant HCC cell lines

MHCC97L and Hep3B cells were grown in T_25_ flasks and treated with saracatinib (2 μmol/L and 1 μmol/L) followed by the addition of increasingly higher concentrations of saracatinib until the MHCC97L cells became stably resistant to 4 μmol/L saracatinib and the Hep3B cells became stably resistant to 2 μmol/L saracatinib. These resistant cells were re-named MHCC97L-Src and Hep3B-Src. Oxaliplatin-resistant HCC cell lines were generated as previously described [3]. MHCC97L cells that were stably resistant to 2 μmol/L oxaliplatin were re-named MHCC97L-Oxa, and Hep3B cells that were stably resistant to 1 μmol/L oxaliplatin were re-named Hep3B-Oxa.

### RNA interference

The siRNA duplexes for *ABCG1* were chemically synthesized by Qiagen, Inc. (Valencia, CA, USA). The following *ABCG1* siRNA sequences were constructed: 5′-CGTGGATGAGGTTGAGACA-3′(forward) and 5′-GGTGGACAACAACTTCACA-3′ (reverse). Chemically synthesized mock siRNA (fluorescein-labeled, non-silencing) was also purchased from Qiagen, Inc. The human full-length cDNA of *ABCG1* were obtained from Genesent (shanghai China) and then cloned into the pCDH lentiviral expression vector (System Biosciences). Using the In-Fusion HD Cloning Kit (Takara), the amplified fragment was inserted into the plasmid pCDH (between XbaI and EcoRI sites). Flag-tagged *ABCG1* in pCDH vector was from Genesent (shanghai China).

### Cell viability assay

Wild-type MHCC97L and Hep3B cells were grown in 96-well plates in medium containing 2 μmol/L oxaliplatin and increasing concentrations of saracatinib for 24, 48, 72, and 96 h. Additionally, wild-type MHCC97L and Hep3B cells were grown in medium containing 2 μmol/L saracatinib and increasing concentrations of oxaliplatin for 24, 48, 72, and 96 h. Cell proliferation assays were performed with CCK8. Results were expressed as absorbance of each well at 450 nm (OD450).

### Animal model and treatment procedures

MHCC97L (5 × 10^6^) were implanted subcutaneously into the upper left flank region of mice to establish subcutaneous xenografts. The synergistic effects of the combination therapy of oxaliplatin (10 mg/kg) and saracatinib (20 mg/kg) were evaluated. Twenty nude mice bearing subcutaneous xenografts were randomly divided into the control, oxaliplatin, saracatinib, and oxaliplatin + sorafenib groups (*n* = 5 per group). Tumor weights were evaluated in 4 weeks after the treatments. Another eighteen nude mice bearing subcutaneous xenografts were randomly divided into the oxaliplatin, oxaliplatin + safacatinib, oxaliplatin + saracatinib + siABCG1 groups (*n* = 6 per group). Small interfering RNA ABCG1 was used for local injection every 10 days. Tumor weights were also evaluated in 4 weeks after the treatments. Intraperitoneal injection of pentobarbital (5 mg/kg) combined with cervical spondylolisthesis was used for the sacrifice of mice after the study.

### Colony formation

To investigate the effect of combination treatment with saracatinib and oxaliplatin on HCC cells, MHCC97L and Hep3B (1 × 10^3^ cells/well) were plated in 6-well plates and cultured with DMEM containing 5% FBS with saracatinib (2 μmol/L) and/or oxaliplatin (2 μmol/L). Culture medium was replaced every 3 d, and the colonies were fixed with ice-cold 4% paraformaldehyde after 14 d. Cells were stained with Giemsa (Sigma, St. Louis, MO, USA) and photographed at × 5 magnification.

### RNA sequencing and bioinformatics analysis

RNA sequencing (Shanghai OE Biotech Co. Ltd., China) was used to compared MHCC97L with MHCC97L-Src and MHCC97L with MHCC97L-Oxa. Principal components analysis (PCA) and hierarchical clustering were performed using the R program. PCA was used to visualize differences between groups. Hierarchical cluster analysis was used to evaluate a set of dissimilarities, serving as a “complete” method for analyzing different genes based on the same gene ontology (GO). The GO seq R package was used to perform GO enrichment analysis of different gene clusters. KEGG enrichment analysis of different gene clusters were implemented using the cluster Profiler R package, and the cutoff for significance was set as *p* = 0.05. Raw sequencing data is publicly available at NCBI (GEO accession number GSE129071).

### Cell cycle assays

MHCC97L cells were starved in serum-free medium for 24 h and then grown in oxaliplatin (2 μmol/L) and/or saracatinib (2 μmol/L) for 48 h. Cell cycle analyses and quantification of genomic DNA fragmentation were performed using the Cell Cycle Detection Kit (KeyGen, Nanjing, China) according to the manufacturer’s protocol. Cell cycle distributions were analyzed by flow cytometry using a Becton Dickinson FACS Calibur (Franklin Lakes, NJ, USA).

### Statistical analysis

A two-sided Student’s *t*-test was performed to evaluate the statistical significance of differences in means. Experiments were performed at least three times, and *p* < 0.05 was considered statistically significant. Statistical analyses were performed using SPSS 15.0 software for Windows (SPSS Inc. Chicago, IL, USA).

## Results

### Combination chemotherapy with oxaliplatin and saracatinib exhibited antagonistic effects

**Fig. 1:**
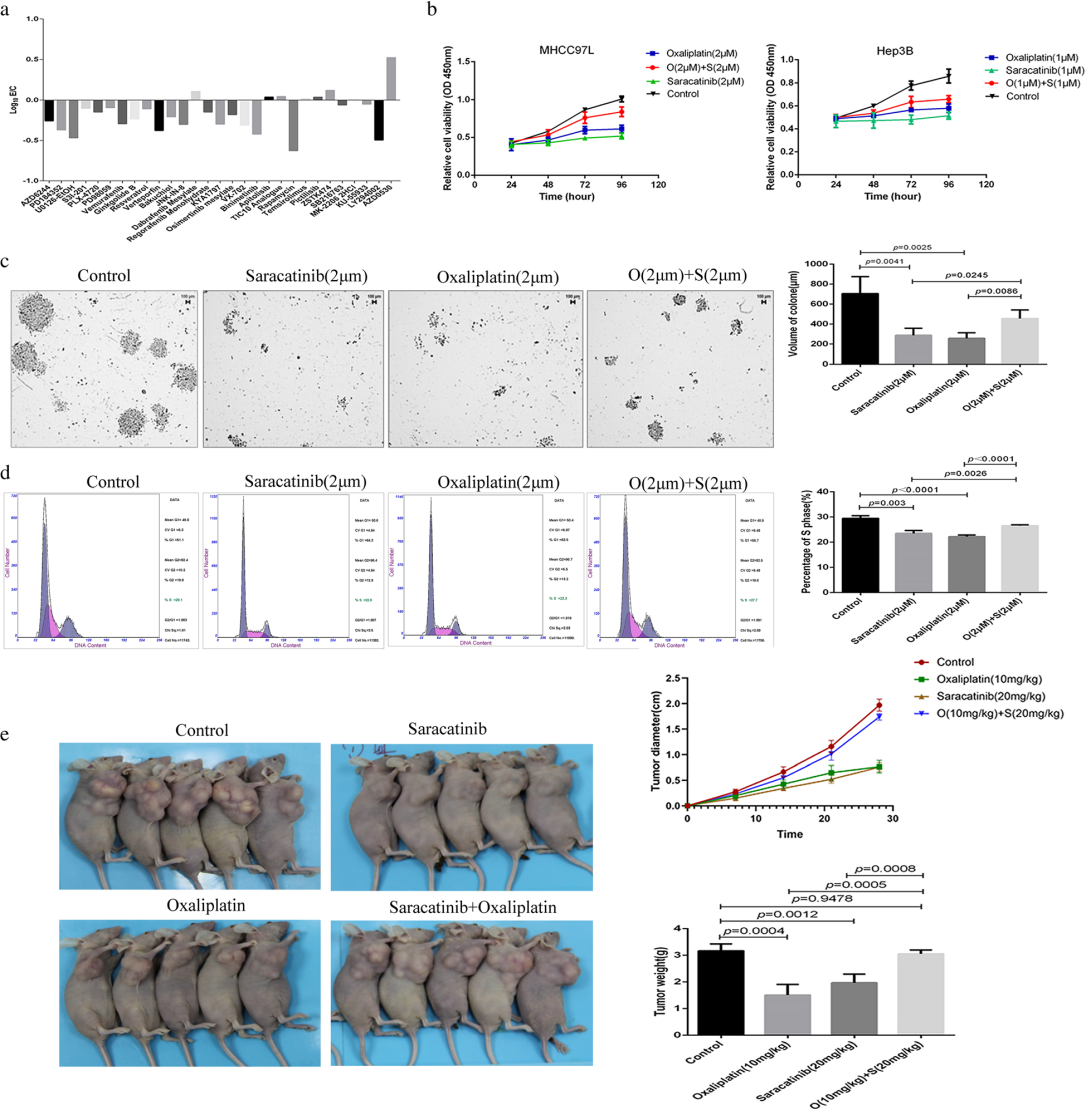
Combination chemotherapy using oxaliplatin and saracatinib induced antagonistic effects on HCC cells. (**a**) The efficacy of combinations between oxaliplatin and anti-cancer molecular targeting drugs was screened, and the combined chemotherapy with oxaliplatin and saracatinib induced significantly antagonistic effects. (**b**) Cell viability assays of MHCC97L and Hep3B cells treated with oxaliplatin or saracatinib individually or in combination. (**c**) Colony formation assays were performed on HCC cells following treatment with either oxaliplatin or saracatinib individually or with both drugs combined. Data are shown as cell colony diameters. (**d**) The cell cycle distributions were analyzed, and the proportions of cells in S phase were determined for HCC cell lines treated with oxaliplatin or saracatinib individually or in combination. (**e**) The tumor weight of the subcutaneous xenografts were larger in the combination treatment group than treatment with the single drugs

The efficacy of combinations between oxaliplatin and anti-cancer molecular targeting drugs was screened. Strangely, the combined chemotherapy with oxaliplatin and saracatinib induced significantly antagonistic effects (Fig. 1a). The HCC cell lines MHCC97L and Hep3B exhibited significant anti-tumor effects with lower proliferation rates than wild-type cells following treatment with oxaliplatin or saracatinib individually; however, combination treatment with the two drugs resulted in a higher proliferation rate, indicating impaired antitumor efficacy with combined treatment (Fig. 1b). To confirm this phenomenon, plate colony formation assays were performed. Compared to untreated cells, colony diameters were significantly decreased following treatment with oxaliplatin (257.51 ± 55.60 μm vs. 705.16 ± 170.81 μm; *p* = 0.041) or saracatinib (287.57 ± 71.36 μm vs. 705.16 ± 170.81 μm; *p* = 0.0025) individually. Combination therapy, on the other hand, resulted in larger colony diameters than oxaliplatin (455.16 ± 86.12 μm vs. 257.51 ± 55.60 μm; *p* = 0.0086) or saracatinib (455.16 ± 86.1 μm vs. 287.25 ± 71.36 μm; *p* = 0.0245) treatment alone (Fig. 1c). Next, we analyzed the cell cycle distributions to further evaluate the observed changes in cell proliferation. The percentages of cells in S phase decreased following treatment with either oxaliplatin (22.321 ± 0.67% vs. 29.48 ± 1.06%; *p*<0.0001) or saracatinib (23.59 ± 1.76% vs. 29.48 ± 1.06%; *p* = 0.003). Combination therapy with oxaliplatin and saracatinib resulted in significantly increased percentages of cells in S phase compared to treatment with oxaliplatin (26.55 ± 0.39% vs. 22.321 ± 0.67%; *p*<0.0001) or saracatinib (26.55 ± 0.39% vs. 23.59 ± 1.76%; *p* = 0.0026) individually (Fig. 1d). Furthermore, the tumor weight of the subcutaneous xenografts were larger in the combination treatment group than oxaliplatin (3.06 ± 0.16 g vs. 1.51 ± 0.39 g; *p* = 0.0005) or saracatinib (3.06 ± 0.16 g vs. 1.97 ± 0.32 μm; *p* = 0.0008) treatment alone (Fig. 1e).

**Fig. 2:**
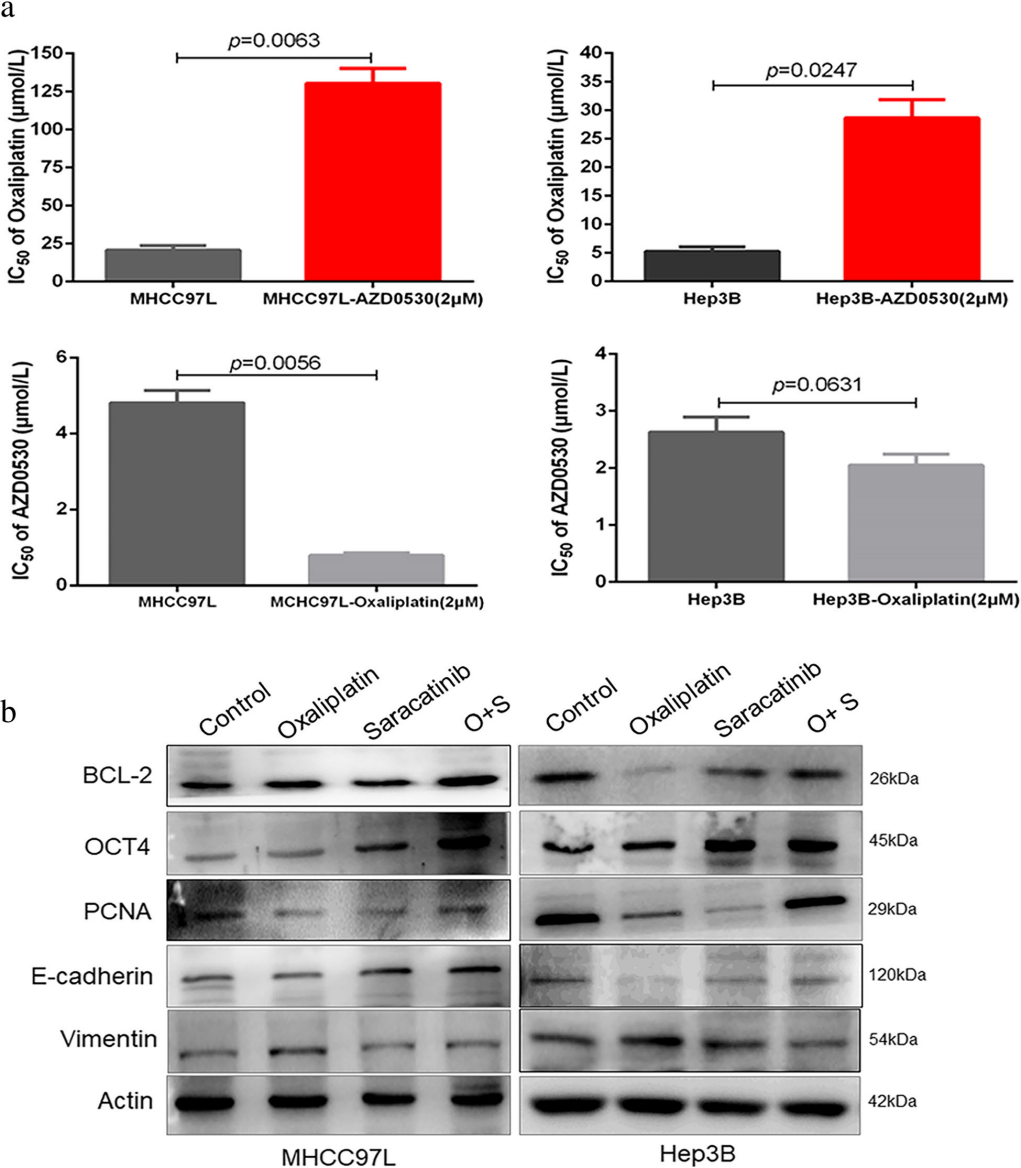
Reconfirmation of the antagonistic effects using oxaliplatin and saracatinib on HCC cells. (**a**) The effects of the combined therapy on the response of HCC cells to different concentrations of chemotherapy drugs were assessed in HCC cell lines via measurement of IC_50_ values. (**b**) The expression of PCNA and EMT-associated biomarkers were detected in HCC cells treated with saracatinib and oxaliplatin individually or in combination

We next revalidated the effects of the combined therapy on the response of HCC cells. MHCC97L (130.6 ± 16.62 μmol/L vs. 20.85 ± 4.86 μmol/L; *p* = 0.0063) and Hep3B (28.67 ± 5.59 μmol/L vs. 5.29 ± 1.29 μmol/L; *p* = 0.0247) cells exhibited significantly increased IC_50_ values in response to oxaliplatin when treated with saracatinib. Interestingly, however, MHCC97L (0.79 ± 0.11 μmol/L vs. 4.81 ± 0.57 μmol/L; *p* = 0.0056) and Hep3B (2.05 ± 0.32 μmol/L vs. 2.62 ± 0.47 μmol/L; *p* = 0.0631) cells exhibited reduced IC_50_ values in response to saracatinib when treated with oxaliplatin as well (Fig. 2a). These findings suggest that saracatinib treatment in combination with oxaliplatin increases oxaliplatin resistance in HCC, and it was the treatment with saracatinib caused oxaliplatin resistance. Additionally, we investigated the effects of the two chemotherapy drugs on protein expression in the two HCC cell lines MHCC97L and Hep3B. Oxaliplatin treatment led to the downregulation of PCNA and the occurrence of EMT, which was associated with the upregulation of vimentin and the downregulation of E-cadherin. Conversely, saracatinib not only downregulated PCNA expression but also reversed the EMT. MHCC97L and Hep3B treated with both oxaliplatin and saracatinib exhibited partial upregulation of PCNA and reversion of EMT (Fig. 2b). These findings suggest that saracatinib treatment in combination with oxaliplatin reduces the antitumor efficacy of these drugs on HCC cells, but reverses the negative effect of EMT induced by oxaliplatin.

### Sequential chemotherapy reduced the antitumor efficacy of oxaliplatin on saracatinib-resistant HCC

**Fig. 3:**
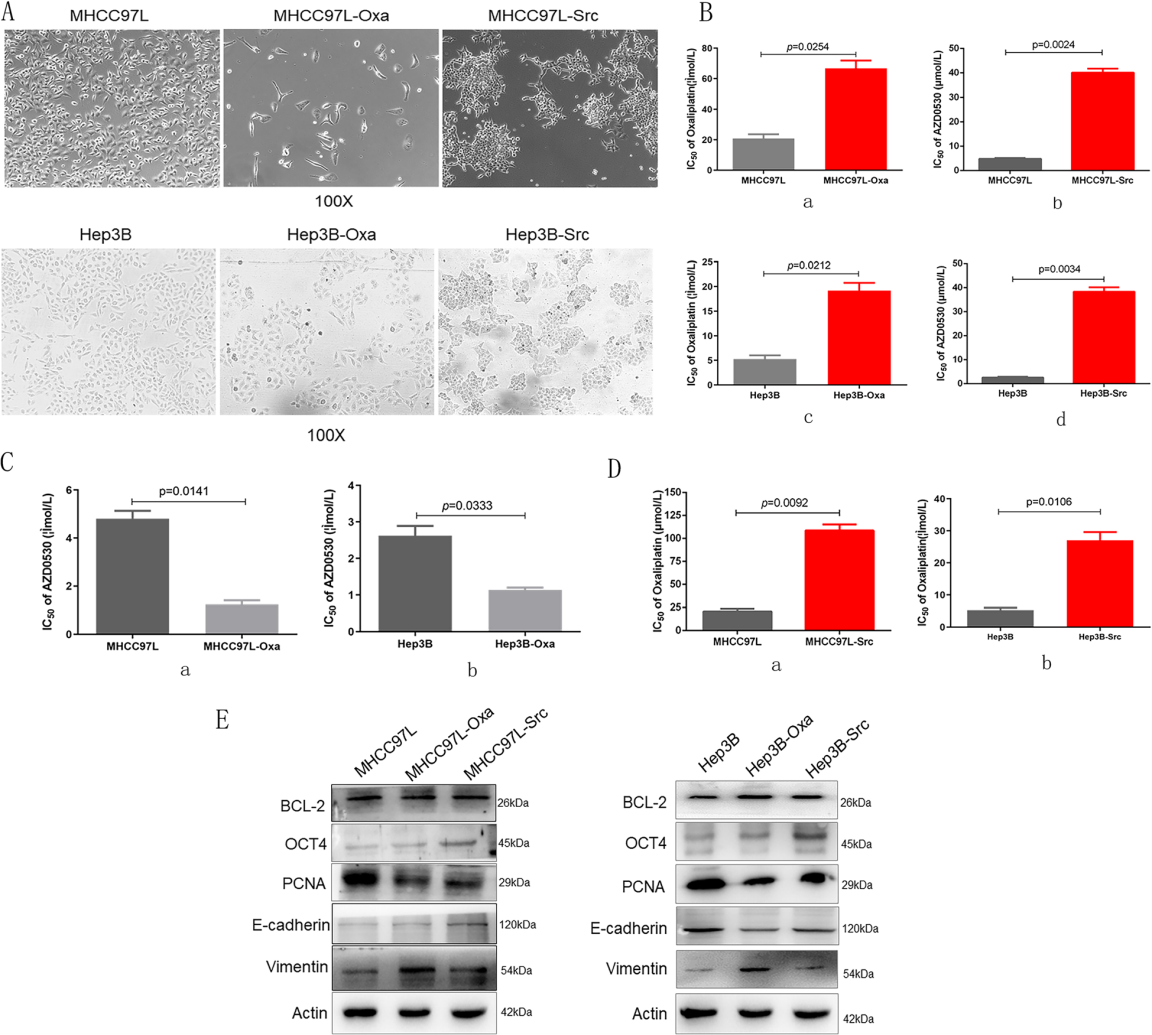
Sequential chemotherapy induced decreased antitumor efficacy of oxaliplatin on saracatinib-resistant HCC. (**A**) Cell morphology was observed in oxaliplatin and saracatinib-resistant HCC cell lines. (**B**) oxaliplatin- and saracatinib resistant HCC cell lines exhibited increased IC_50_ values to oxaliplatin and saracatinib separately. (**C**) Chemoresistance of oxaliplatin-resistant HCC cell lines to saracatinib was determined with decreased IC_50_ values compared with wild-type HCC cells. (**D**) Saracatinib-resistant HCC cell lines exhibited significantly enhanced resistance to oxaliplatin with an increased IC_50_ to oxaliplatin. (**E**) Expression of proteins related to proliferation and EMT in oxaliplatin- and saracatinib-resistant cell lines were examined by immunoblotting

In order to simulate the clinical sequential chemotherapy, HCC cell lines were treated continually with oxaliplatin to generate oxaliplatin-resistant cell lines (MHCC97L-Oxa and Hep3B-Oxa) that exhibited decreased intercellular adhesion and spindle-shaped cell morphology (Fig. 3A). Compared to wild-type HCC cells, MHCC97L-Oxa (66.67 ± 9.01 μmol/L vs. 31.67 ± 4.04 μmol/L; *p* = 0.0254; Fig. 3B, a) and Hep3B-Oxa (19.21 ± 2.69 μmol/L vs. 5.45 ± 1.23 μmol/L; *p* = 0.0212; Fig. 3B, c) exhibited increased oxaliplatin IC_50_ values. The oxaliplatin-resistant HCC cell lines were next treated with increasing concentrations of saracatinib, resulting in a decrease of IC_50_ values in MHCC97L-Oxa (1.23 ± 0.31 μmol/L vs. 4.30 ± 0.97 μmol/L; *p* = 0.0141; Fig. 3C, a) and Hep3B-Oxa (1.14 ± 0.11 μmol/L vs. 2.62 ± 0.47 μmol/L; *p* = 0.0333; Fig. 3C, b) compared to the parental wild-type HCC cells, still exhibiting more sensitive to saracatinib.

Saracatinib-resistant MHCC97L and Hep3B cells (MHCC97L-Src and Hep3B-Src) were generated similarly via continuous treatment with saracatinib, and these lines exhibited enhanced intercellular adhesion and appeared as agglomerated cell clumps (Fig. 3A). Compared to wild-type cells, MHCC97L-Src (40.07 ± 2.88 μmol/L vs. 4.81 ± 0.57 μmol/L; *p* = 0.0024; Fig. 3B, b) and Hep3B-Src (38.36 ± 3.17 μmol/L vs. 2.62 ± 0.47 μmol/L; *p* = 0.009; Fig. 3B, d) exhibited increased saracatinib IC_50_ values. The saracatinib-resistant HCC cell lines were then treated with oxaliplatin at increasing concentrations, yielding significantly enhanced resistance to oxaliplatin with increased IC_50_ in both MHCC97L-Src (108.71 ± 11.24 μmol/L vs. 20.85 ± 4.86 μmol/L; *p* = 0.0092; Fig. 3D, a) and Hep3B-Src (27.01 ± 4.59 μmol/L vs. 5.29 ± 1.29 μmol/L; *p* = 0.0106; Fig. 3D, b) compared to wild-type cells.

Additionally, we investigated protein expression in oxaliplatin- and saracatinib-resistant cell lines. MHCC97L-Oxa and Hep3B-Oxa exhibited downregulation of PCNA and E-cadherin and upregulation of vimentin and OCT4, while MHCC97L-Src and Hep3B-Src exhibited downregulation of PCNA, upregulation OCT4, and reversion of EMT compared to the parental cell lines (Fig. 3E). Together, these results indicate that sequential chemotherapy reduced the antitumor efficacy of oxaliplatin on saracatinib-resistant HCC.

### ABCG1 upregulation and Wnt signaling pathway activation are integral mechanisms involved in the antagonism between saracatinib and oxaliplatin in HCC

**Fig. 4:**
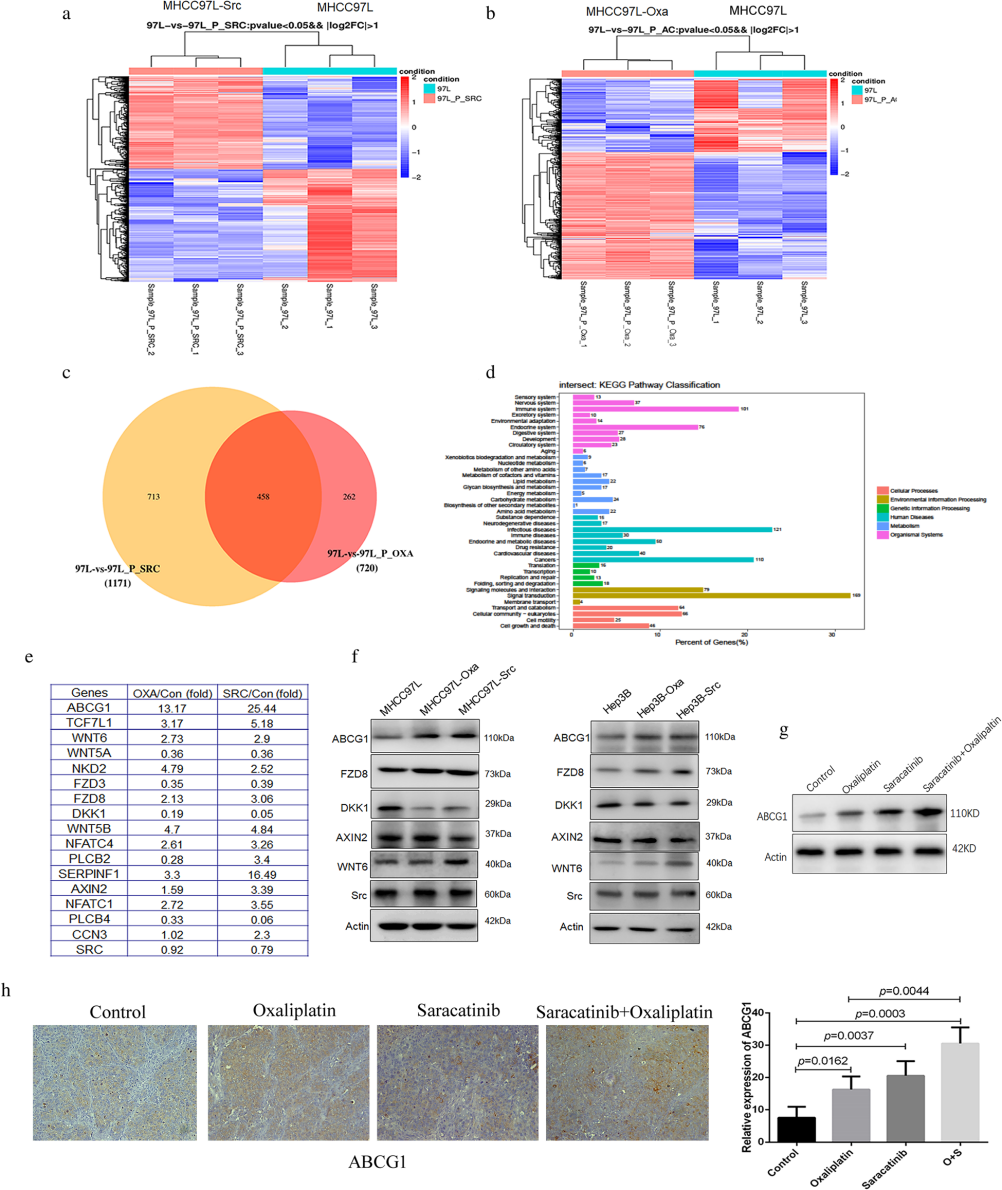
Gene expression profiles in saracatinib- and oxaliplatin-resistant HCC revealed upregulation of ABCG1 and activation of Wnt signaling-associated proteins. Gene expression profiles were compared between wild-type MHCC97L and MHCC97L-Src (**a**) and between MHCC97L and MHCC97L-Src (**b**). (**c**) A total of 458 genes were found to be altered in both sets of cells. (**d**) These genes were related to cell division, growth, angiogenesis, adhesion, and metabolic processes, and KEGG pathway analysis was performed on these altered genes. (**e**) The altered genes related to drug resistance were partly selected. (**f**) Immunoblotting was used to determine the protein expression of the genes that were altered most dramatically between the wild-type and drug-resistant cell lines. (**g**) Immunoblotting confirmed the upregulation of ABCG1 after combination treatment with oxaliplatinor and saracatinib. (**h**) Immunohisochemotherapy verified that the expression of ABCG1 was significantly upregulated after the treatment with oxaliplatinor or saracatinib and combination treatment in subcutaneous xenografts tissues

The expression of 20,030 genes was compared between wild-type MHCC97L and MHCC97L-Src cells in three independent experiments (Fig. 4a). Gene expression profiles for 1172 genes exhibited differences (*p* < 0.05) between MHCC97L and MHCC97L-Src, implicating these genes in saracatinib resistance. Furthermore, analysis of these associated “resistance” genes revealed 526 upregulated and 645 downregulated genes. The expression of these 20,300 genes in wild-type MHCC97L and MHCC97L-Oxa was also compared (Fig. 4b). Expression profiles for 720 genes exhibited differences (*p* < 0.05) between MHCC97L and MHCC97L-Oxa, implicating these genes in oxaliplatin resistance. Of these, 455 were upregulated, and 265 were downregulated in the two drug-resistant cell lines.

A total of 458 altered genes overlapped between the two drug-resistant cell lines, and all of these were closely related to cell division, growth, angiogenesis, adhesion, and metabolic processes (Fig. 4c). KEGG pathway analysis revealed that 20 of the altered genes were related to drug resistance: *ABCG1*, *ATM*, *BBC3*, *BIK*, *BIRC3*, *CDKN1A*, *DLL4*, *ERBB3*, *FGF2*, *FOS*, *GPER1*, *IL6*, *JAG1*, *MMP2*, *NRG2*, *PDGFRB*, *PIK3CA*, *SHC4*, *TOP2A*, *TOP2B*, and *VEGFA.* Another 16 genes were related to Wnt signaling: *ROCK2*, *TCF7L1*, *WNT6*, *WNT5A*, *NKD2*, *FZD3*, *FZD8*, *DKK1*, *WNT5B*, *NFATC4*, *PLCB2*, *SERPINF1*, *AXIN2*, *NFATC1*, *PLCB4*, and *RAC2*. Immunoblotting confirmed the upregulation of ABCG1 and Wnt-associated proteins, including FZD8, DKK1, Axin2, and WNT6 (Fig. 4d, e, f and g). Immunohisochemotherapy verified that the expression of ABCG1 was significantly upregulated after the treatment with oxaliplatin (16.25 ± 4.03 vs. 7.50 ± 3.42 μmol/L; *p* = 0.0162) or saracatinib (20.50 ± 4.51 vs. 7.50 ± 3.42 μmol/L; *p* = 0.0037) in subcutaneous xenografts tissues. And the combination treatment exhibited higher expression of ABCG1 than oxaliplatin single use (30.50 ± 5.01 vs. 16.25 ± 4.03; *p* = 0.0044; Fig. 4h). Therefore, we speculate that ABCG1 upregulation and Wnt signaling pathway activation are integral mechanisms involved in the antagonism between saracatinib and oxaliplatin in HCC.

### Interference with *ABCG1* expression or inhibition of Wnt signaling resulted in reversal of the saracatinib-induced oxaliplatin resistance in HCC

**Fig. 5:**
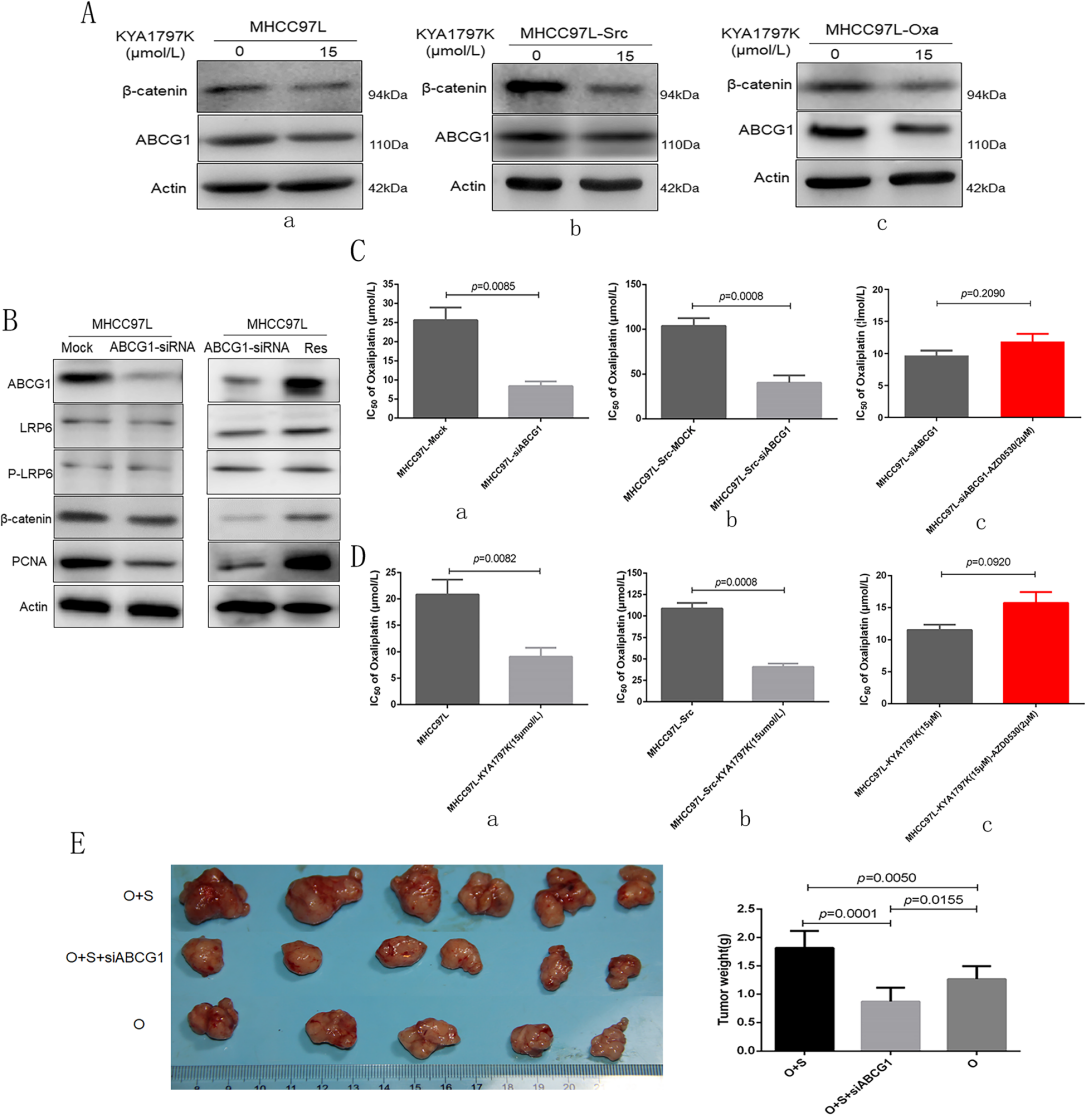
Upregulation of ABCG1 via alterations in Wnt/β-catenin signaling contributes to the effects of saracatinib on oxaliplatin resistance of HCC cells. (**A**) Immunoblotting was used to examine the expression of ABCG1 following Wnt/β-catenin signaling pathway inhibition by KYA1797K. (**B**) Wnt/β-catenin signaling pathway and PCNA expression was determined following silencing of ABCG1 by siRNA and restoration treatment in HCC cells. (**C**) The sensitivity of HCC cells to oxaliplatin was measured as IC_50_ following silencing of ABCG1 expression with siRNA or mock siRNA treatment. (**D**) The sensitivity of cells to oxaliplatin following treatment with the Wnt signaling inhibitor KYA1797K was determined as IC_50_ values. (**E** ) Interference with *ABCG1* expression resulted in reversal of the saracatinib-induced oxaliplatin resistance with smaller subcutaneous xenografts

Immunoblotting verified that the expression of ABCG1 was significantly downregulated by Wnt/β-catenin signaling pathway inhibition with KYA1797K in wild-type HCC cell lines, MHCC97L-Src, and Hep3B-Src (Fig. 5A). Following ABCG1 downregulation and, the key cell membrane receptors for Wnt signaling LRP6 and p-LRP6 were not significantly altered; however, β-catenin was slightly downregulated, and the expression of PCNA was significantly decreased. ABCG1 restoration could reverse this alteration in protein levels (Fig. 5B).

Next, we confirmed the role of ABCG1 and Wnt signaling in oxaliplatin resistance. ABCG1 was silenced using specific siRNA in MHCC97L cells, resulting in a decreased IC_50_ to oxaliplatin compared to mock-treated MHCC97L cells (MHCC97L-Mock; 8.41 ± 2.09 μmol/L vs. 25.59 ± 5.82 μmol/L; *p* = 0.0085; Fig. 5C, a). Following silencing of ABCG1 using siRNA in saracatinib-resistant MHCC97L (MHCC97L-Src-ABCG1-Sh1), we observed decreased resistance to oxaliplatin (40.43 ± 8.12 μmol/L vs. 103.71 ± 8.74 μmol/L; *p* = 0.0008; Fig. 5C, b) compared to mock-treated cells. Furthermore, silencing of ABCG1 combined with saracatinib in MHCC97L, there was no significantly increased resistance to oxaliplatin (11.84 ± 2.11 vs. 9.73 ± 1.26 μmol/L; *p* = 0.0874; Fig. 5C, c). Additionally, HCC cells treated with the Wnt signaling inhibitor KYA1797K exhibited decreased resistance to oxaliplatin with reduced IC_50_ values (11.07 ± 2.02 μmol/L vs. 31.67 ± 4.04 μmol/L; *p* = 0.0082; Fig. 5D, a). Treatment of MHCC97L-Src cells with KYA1797K resulted in decreased resistance to oxaliplatin (40.83 ± 8.12 μmol/L vs. 108.71 ± 11.24 μmol/L; *p* = 0.0008; Fig. 5D, b). KYA1797K combined with saracatinib in MHCC97L, there was no significantly increased resistance to oxaliplatin (11.51 ± 1.44 vs. 15.73 ± 2.95 μmol/L; *p* = 0.0920; Fig. 5D, c).

Furthermore, it was confirmed once again that the tumor weight of the subcutaneous xenografts was larger in combination treatment group using oxaliplatin and saracatinib than oxaliplatin single use (1.82 ± 0.30 g vs. 1.26 ± 0.23 g; *p* = 0.0050; Fig. 5E). And the subcutaneous xenografts was smaller in the combination treatment group using oxaliplatin, saracatinib, and ABCG1 siRNA local injection (0.87 ± 0.24 g vs. 1.82 ± 0.30 g; *p* = 0.0001; Fig. 5E) than combination group only using oxaliplatin and saracatinib. Together, these findings suggest that ABCG1 and Wnt signaling contribute to oxaliplatin resistance in saracatinib-treated HCC cells. And interference with *ABCG1* expression or inhibition of Wnt signaling resulted in reversal of the saracatinib-induced oxaliplatin resistance in HCC.

## Discussion

Liver cancer, most commonly seen as hepatocellular carcinoma (HCC), has high prevalence and incidence rates in China, which accounts for more than 50% of the total number of liver cancer cases and deaths in the world [4]. One of the chemotherapeutic drugs for patients with advanced HCC is oxaliplatin, which initiates apoptosis by inhibiting the replication and transcription of DNA in HCC cells [5]. However, the efficacy of oxaliplatin on HCC is poor, exhibiting intrinsic and acquired resistance. Therefore, methods to enhance oxaliplatin treatment responses are urgently needed. In this study, the efficacy of combinations between oxaliplatin and anti-cancer molecular targeting drugs was screened, and saracatinib treatment actually induced resistance to oxaliplatin treatment was proved.

In many solid tumor cells, including HCC, Src expression level or activity is increased [6–8], promoting metastasis [9, 10]. Activation of the tyrosine kinase Src is responsible for tumor progression promoted by insulin-like growth factor 1 receptor (IGF-1R) [11, 12]. Previously, we demonstrated that high expression of IGF1 was closely associated with the maintenance of stemness in oxaliplatin-resistant HCC cells and that IGF1-IGF1R signaling blockade effectively increased oxaliplatin sensitivity [3]. Tyrosine kinase Src was discovered more than 30 years ago as a kinase that is involved in the crosstalk between many signaling pathways, including the integrin/FAK, Ras/Raf/MEK, PI3K/AKT, and IGF1/IGF1R pathways, and Src activation promotes cell proliferation, adhesion, invasion, migration, metastasis, and tumorigenesis [13]. Recently, Liu et al. [14] reported that increased expression of Src potentiates ERK activation and reverses sorafenib resistance in HCC. Thus, inhibition of Src may provide a new strategy for drug combination studies for HCC treatment [15]. Based on these findings, we expect a synergistic relationship between oxaliplatin and Src kinase inhibition. Saracatinib (AZD0530) is a potent, orally administered small molecule that inhibits Src by blocking the ATP binding site of the kinase [16]. However, the combined chemotherapy with oxaliplatin and saracatinib induced significantly antagonistic effects. Recent research proved saracatinib failed to demonstrate monotherapeutic efficacy, with undesirable stem cell-promoting functions in patients with head and neck squamous cell carcinoma [17]. In the present study, we proved the combined treatment of HCC with oxaliplatin and saracatinib impaired the efficacy of either drug individually. And it was mainly saracatinib treatment increased oxaliplatin resistance in HCC. We also tested the effects of the sequential treatment of HCC with oxaliplatin and saracatinib, and sequential chemotherapy also reduced the antitumor efficacy of oxaliplatin on saracatinib-resistant HCC.

Based on previous and our current findings, oxaliplatin-resistant HCC cells exhibited decreased intercellular adhesion and spindle-shaped cell morphology that are characteristic of EMT [18], while saracatinib-resistant HCC cells exhibited enhanced intercellular adhesion and cell clumping [19]. Immunoblotting further confirmed that oxaliplatin treatment led to the occurrence of EMT as vimentin was upregulated and E-cadherin was downregulated. Saracatinib significantly inhibited the expression of PCNA and reversed the EMT. Gene expression analysis revealed 458 genes that were altered in both saracatinib- and oxaliplatin-resistant HCC, and these genes were related to processes of cell division, growth, angiogenesis, adhesion, and metabolism. Based on KEGG pathway classification, 20 of these genes were related to drug resistance, while 16 were related to Wnt signaling activation. Furthermore, immunoblotting revealed that ABCG1 and the Wnt/β-catenin signaling pathway were both upregulated in HCC cell lines in the presence of continuous treatment with saracatinib or oxaliplatin.

ABCG1 is a cholesterol lipid efflux pump that plays a well-known role in tumor growth, conferring chemoresistance to various malignant tumors [20]. Several ABC transporters, including ABCG1, are associated with multidrug resistance (MDR), which is a major obstacle to the effective clinical treatment of cancer [21]. In the present study, ABCG1 is a downstream protein in Wnt/β-catenin signaling and can be significantly downregulated by Wnt/β-catenin signaling pathway. Interference with the expression of ABCG1 or inhibition of Wnt/β-catenin results in decreased oxaliplatin resistance, supporting a role for these proteins in the acquired drug resistance of HCC. Understanding the molecular pathogenesis of HCC chemoresistance is key to improving patients’ prognosis.

## Limitation

In the present study, we demonstrated that combined or sequential chemotherapy with oxaliplatin and saracatinib induced antagonistic effects, while our study was only limited to liver cancer, and lack of deeper mechanistic knowledge of the actions of saracatinib. Therefore, several fundamental questions remain to be answered concerning the combined or sequential chemotherapy with the two drugs in the further study. (1) Whether antagonisms are limited to a few specific cancer species? (2) Deeper mechanisms involved in antagonistic effects of the two drugs are also needed be explored? (3) Can saracatinib be used in other diseases is also need to be answered? Still, our results do provide some important clues that may help guide drug selection and therapeutic strategy used in clinical treatments of cancer.

## Conclusions

From our experimental results and our review of the literature, we propose the following conclusions. (1) The combined and sequential chemotherapy with oxaliplatin and saracatinib induces significantly antagonistic effects. (2) ABCG1 upregulation and Wnt signaling pathway activation are integral mechanisms involved in the antagonism between saracatinib and oxaliplatin in HCC. (3) Interference with ABCG1 expression or inhibition of Wnt signaling resulted in reversal of the saracatinib-induced oxaliplatin resistance in HCC.

## Data Availability

All data generated or analyzed during this study are included in this published article. The datasets used and/or analyzed and materials developed during the current study are available from the corresponding author by reasonable request.

## Abbreviations

CCK8: Cell Counting Kit 8
DMEM: Dulbecco’s Modified Eagle’s Medium
FBS: Fetal bovine serum
GO: Gene ontology
HCC: Hepatocellular carcinoma
IGF-1R: Insulin-like growth factor 1 receptor
MDR: Multidrug resistance
PCA: Principal components analysis
TACE: Transcatheter arterial chemoembolization
